# Pseudomicroangiopathic Thrombotic Syndrome: Unveiling the Vitamin B12 Deficiency Connection

**DOI:** 10.7759/cureus.61787

**Published:** 2024-06-06

**Authors:** Samia Sabri, Zahida Aqodad, Habiba Alaoui, Houda Bachir, Siham Hamaz, Khalid Serraj

**Affiliations:** 1 Department of Internal Medicine, Immuno-Hematology and Cellular Therapy Laboratory, Faculty of Medicine and Pharmacy, University Hospital Mohammed Vl, Mohammed First University, Oujda, MAR

**Keywords:** microangiopathic, anemia, biermer, vit b12 deficiency, vit b12

## Abstract

Pernicious anemia, a manifestation of vitamin B12 deficiency, can present with a spectrum of hematological abnormalities, sometimes mimicking more severe conditions such as thrombotic microangiopathy (TMA). This case report details a 53-year-old female who presented with significant weight loss, watery diarrhea, and jaundice. Laboratory investigations revealed pancytopenia, hemolysis, and schistocytes, initially suggesting a diagnosis of microangiopathic hemolytic anemia (MAHA). However, significantly low vitamin B12 levels and subsequent bone marrow examination confirmed pernicious anemia with megaloblastic changes. This case underscores the importance of considering vitamin B12 deficiency in the differential diagnosis of patients presenting with TMA-like symptoms. Early recognition and treatment with vitamin B12 supplementation led to rapid clinical improvement and the resolution of symptoms. This report highlights the need for heightened clinical awareness of atypical presentations of pernicious anemia to prevent misdiagnosis and ensure timely, effective treatment.

## Introduction

Vitamin B12 deficiency is a common and potentially serious condition. Clinical manifestations are often initially subtle and develop insidiously [1-4]. These manifestations include sensory polyneuropathy, isolated hematologic abnormalities such as macrocytosis or hypersegmentation of neutrophils, and potentially severe conditions such as combined spinal cord degeneration or hemolytic anemia. In extreme cases, patients may present with pancytopenia and pseudo-thrombotic microangiopathy (TMA) [1,5].

## Case presentation

A 53-year-old female with no significant past medical history was admitted to the emergency department with complaints of significant weight loss and watery diarrhea lasting for 10 days. On physical examination, the patient had stable hemodynamics, presented with asthenia, and had non-bloody, non-mucoid liquid diarrhea. Additionally, she exhibited noticeable jaundice without tumor syndrome or hemorrhage.

Laboratory investigations revealed the following results (Table 1).

**Table 1:** Laboratory results.

Parameter	Result	Normal Range
Hemoglobin (Hb)	5.1 g/dL	12-16 g/dL
Platelet Count	62,000/mm³	150,000-450,000/mm³
Neutrophil Count	1,050/mm³	1,500-8,000/mm³
Mean Corpuscular Volume (MCV)	113 fL	80-100 fL
Reticulocyte Count	92,000/mm³	>120,000/mm³
Prothrombin Time (PT)	44%	70-100%
Renal Function	Normal	Normal
Haptoglobin	Decreased	30-200 mg/dL
Free Bilirubin	31 mmol/L	0-20 mmol/L
Lactate Dehydrogenase (LDH)	1,466 IU/L	120-240 IU/L
Schistocytes	2.3%	0%
Direct Coombs Test	Positive (IgG)	Negative
Vitamin B12	50 pg/mL	200-900 pg/mL

**Figure 1:**
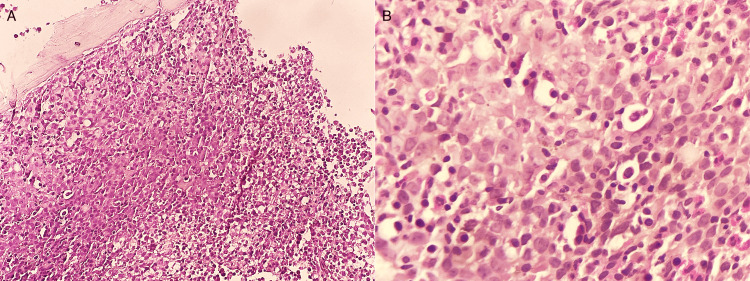
Bone marrow examination showed (A) H&E x 100 showing marked hypercellularity with increased erythroblasts and (B) H&E x400 showing megaloblasts with fine chromatin and marked nucleoli.

**Figure 2:**
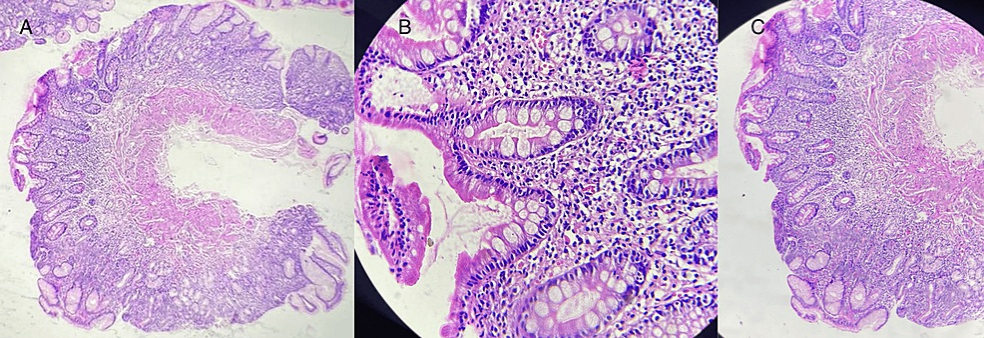
Histological examination of gastrointestinal endoscopy biopsies showing (A) atrophy of the fundic mucosa and (B-C) intestinal metaplasia.

The combination of anemia, thrombocytopenia, and hemolysis with schistocytes raised concerns for a potential diagnosis of microangiopathic hemolytic anemia (MAHA); however, the absence of renal or neurological impairment was atypical. Vitamin B12 levels were found to be significantly low at 50 pg/mL. Given the pancytopenia and atypical hemolytic anemia, a bone marrow examination was performed, revealing megaloblastic changes (Figure 1). Further testing, including anti-parietal cell antibodies and gastroscopy, confirmed the diagnosis of pernicious anemia (Biermer's disease) with fundic atrophy (Figure 2).

The patient's clinical course showed significant improvement following vitamin B12 supplementation, with diarrhea resolving by day four and a reticulocyte crisis by day seven, without the need for additional therapeutic interventions.

## Discussion

Although typically benign, vitamin B12 deficiency can have severe consequences, particularly because of its potential clinical atypicality, which threatens prognosis and can lead to significant diagnostic delays. These delays sometimes result in dramatic and irreversible neurological manifestations and/or lesions [4]. Our case underscores this point and adds to the body of literature documenting atypical presentations of cobalamin deficiency.

The classic clinical presentation of vitamin B12 deficiency is macrocytic, nonregenerative anemia. However, more severe presentations, including pancytopenia with signs of hemolysis, can occur in cases of profound deficiency, mimicking TMA. In such cases, it is crucial to rule out thrombotic thrombocytopenic purpura (TTP), a condition associated with high mortality. The use of the PLASMIC score helps stratify the risk of ADAMTS13 (disintegrin and metalloproteinase with thrombospondin motifs 13) deficiency and decide between plasma exchange therapy and simple vitamin B12 supplementation [4,6].

Given the low risk of TTP in our patient (PLASMIC score of 4, the PLASMIC score stands for **p**latelet count/**l**actate dehydrogenase (LDH) level/**a**cute kidney injury/**s**chistocytes/**m**ean corpuscular volume (MCV)/**i**nternational normalized ratio (INR)/**c**linical history of cancer or solid organ transplant, we opted for vitamin B12 supplementation without resorting to plasma exchange therapy. In the study by Federici et al., vitamin B12 deficiency was revealed by pseudo-TMA in 2.5% of cases and by hemolytic anemia in 1.5% of cases [6].

A regenerative hemolytic syndrome can be observed in cases of bone marrow failure (e.g., vitamin deficiency, myelodysplastic syndrome) and chronic hemolysis with folate deficiency. The positivity of the direct Coombs test for IgG is notable. This positivity does not necessarily indicate reduced erythrocyte survival and can result from technical errors, nonspecific binding, hypergammaglobulinemia, or administration of intravenous immunoglobulins (IVIG). Several studies in the literature have found an association between pernicious anemia and a positive Coombs test. The positivity of this test could support the hypothesis of a dysimmune etiology in this vitamin deficiency [7].

## Conclusions

In summary, this case underscores the importance of considering atypical presentations of pernicious anemia, which can mimic severe hematological conditions. Prompt recognition and treatment with vitamin B12 supplementation are crucial for preventing complications and improving patient outcomes. Heightened clinical suspicion and timely intervention are essential in managing such cases effectively and mitigating the risk of irreversible neurological damage.
